# HIV anti-latency treatment mediated by macromolecular prodrugs of histone deacetylase inhibitor, panobinostat†
†Electronic supplementary information (ESI) available. See DOI: 10.1039/c5sc03257a


**DOI:** 10.1039/c5sc03257a

**Published:** 2016-01-05

**Authors:** Kaja Zuwala, Anton A. A. Smith, Martin Tolstrup, Alexander N. Zelikin

**Affiliations:** a Department of Chemistry , Aarhus University , Aarhus , Denmark . Email: zelikin@chem.au.dk; b Department of Infectious Diseases , Aarhus University Hospital , Denmark . Email: marttols@rm.au; c iNano Interdisciplinary Nanoscience Centre , Aarhus University , Aarhus , Denmark

## Abstract

Histone deacetylase inhibitors (HDACi) and panobinostat in particular are currently in the focus of intensive investigation as latency reversing agents against the human immunodeficiency virus (HIV). Regretfully, HDACi have dose limiting side-effects making controlled, optimized methods for delivery of panobinostat highly warranted. This has proven to be highly challenging, predominantly because panobinostat has no readily available classic sites for bioconjugation. In this work, we address this challenge and present the first macromolecular prodrugs of panobinostat engineered using self immolative linkers (SIL) and a disulfide trigger for drug release upon cell entry. Synthetic methodology involved the development of a novel monomer with functionalities of SIL and activated ester for one-step polymer-analogous conjugation to drugs. In agreement with the design set forward, copolymers were stable in buffered solutions and released panobinostat at reducing conditions. Synthesized polymers were highly efficacious as latency reversing agents as monitored in three cell lines harboring latent HIV, at no expense to the cytotoxicity of treatment. The data presented herein provide broad pre-*in vivo* characterization of a promising prodrug system developed to address a global healthcare challenge, safe and efficient reversal of HIV latency.

## Abstract 2

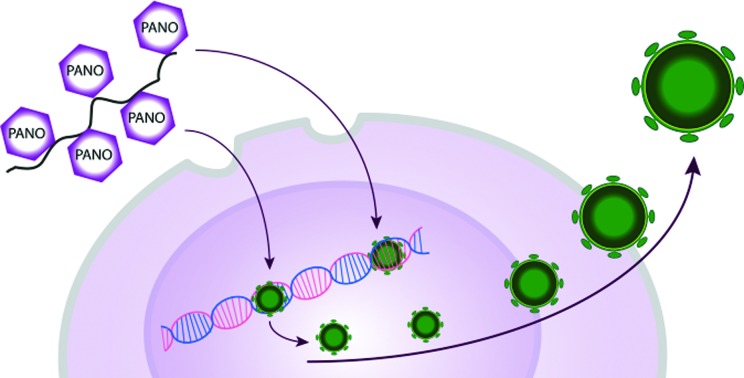
Self-immolative linker and a disulfide trigger are used to design macromolecular prodrugs of panobinostat as efficacious HIV latency reversing agents.

## 

We report the first macromolecular agents engineered as latency reversing agents (LRA) against the human immunodeficiency virus (HIV). HIV forms a chronic, incurable infection that can be managed with antiretroviral therapy (ART). However, the early establishment of a latent reservoir necessitates lifelong ART to suppress HIV replication and prevent disease progression.^1^,^2^ Living with a chronic infection and taking daily medication has tremendous impact on the quality of life of infected individuals and is also of tremendous socio-economic burden. Thus, the development of a cure for HIV is being heavily pursued. One approach is the “shock and kill” where the latent state of the viral infection is “shocked” by an LRA.^1^,^2^ The shock is believed to unmask the virally infected cells to the immune system. Histone deacetylase inhibitors (HDACi) are investigated as possible LRA and we recently published results of a proof-of-concept clinical phase I/II study of 8 week panobinostat (PANO, an HDACi) treatment in ART-treated HIV infected patients.^3^ However, the broader use of these agents requires careful monitoring in that systemic distribution of HDACi may lead to uncontrollable activation of undesired genomic regions and long term usage is often accompanied by thrombocytopenia and neutropenia – which are the dose limiting side effects. Higher doses of PANO are toxic and recently, PANO was granted an FDA approval as an anticancer medication.^4^

One possible approach to minimize systemic side effects is to extend the residence of PANO in the blood (passive targeting) or engineer chemical methods to bring PANO to the target cells (active targeting). This can be accomplished using macromolecular prodrugs (MP)^5^–^7^ and/or antibody–drug conjugates (ADC),^8^,^9^ that is, macromolecular agents. Either case relies on the development of facile bioconjugation and engineered mechanisms of drug release.^10^ However, the chemistry of PANO does not present typical handles for bioconjugation and creation of biodegradable linkages (such as an amine or a thiol functionality). To our knowledge, there are no literature reports on ADC or MP of PANO. In this work, we specifically address this challenge and develop MP of PANO equi-efficacious to the parent drug in activating latent HIV as studied in three different cell lines, providing a sound *in vitro* evaluation of these prodrugs.

Therapeutic benefit delivered by MP is well celebrated in the anti-cancer treatment whereby high molar mass of the polymer–drug conjugates leads to an increased hydrodynamic radius of the molecule, enhanced blood residence time as compared to the parent drug, and accumulation of the conjugate in the tumour *via* the enhanced permeation–retention effect (EPR).^5^,^6^ Prolonged circulation times are also highly beneficial for delivery of anti-HIV medication. Thus, short half-life of the drugs can result in too low exposure and subsequently lead to development of drug resistance.^11^ In contrast, extended blood residence time provides a sufficient concentration of the circulating drug even in case of non-adherence and extended time between drug dosing.^12^ For PANO, the main obstacle to this is that chemistry of this molecule does not present the “classic” handles for bioconjugation and creation of biodegradable linkages (Fig. 1A). To solve this chemical incompatibility, we propose to use self-immolative linkers (SIL), a recently developed cunning tool of organic chemistry.^13^ With the view to achieve a triggered intracellular drug release, we used a thiol-containing SIL and conjugation to polymers *via* a disulfide linkage.^14^ The latter is among the most popular tools of bioconjugate chemistry and releases the drug once inside the cell *via* the glutathione-triggered thiol-disulfide exchange.^15^

**Fig. 1:**
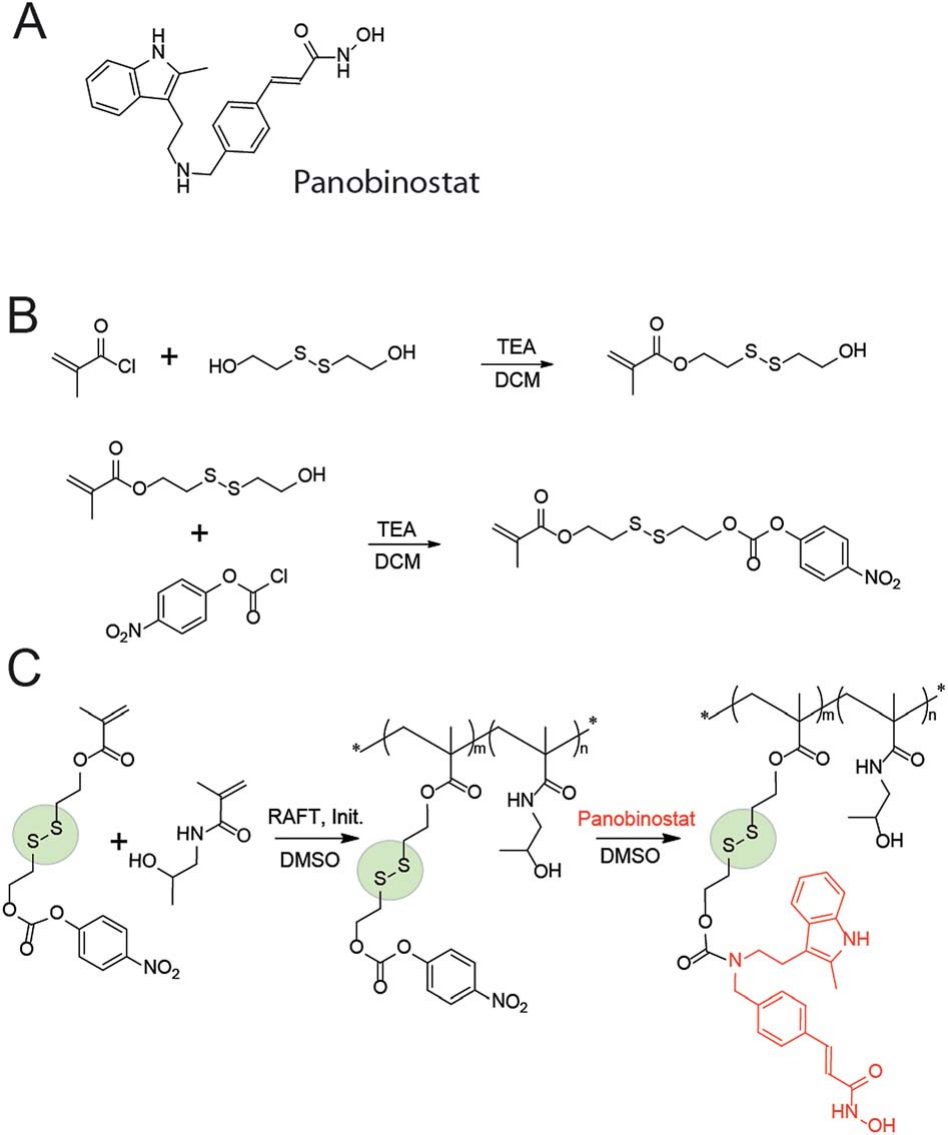
Chemical structure of panobinostat (A); schematic illustration of the synthesis of the activated SIL monomer (B), copolymerization with HPMA and post-functionalization into the panobinostat MPs (C).

MP were engineered based on a polymer with a documented characterization in polymer therapeutics and clinical evaluation, poly(*N*-2-hydroxypropyl methacrylamide), PHPMA.^16^ For bioconjugation, we designed a monomer with a functionality of activated ester connected to the methacrylate *via* a spacer comprised of a disulfide linkage and an SIL, Fig. 1B. Attractive aspects of this monomer are the ease of its synthesis and its capability of forming SIL to drugs with diverse structure, prior to or after the polymerization. In contrast to the existing “activated esters” or similar monomers designed towards conjugation of drugs to carrier (bio)polymers,^17^ this monomer has a built-in disulfide linkage – for a specific intracellular drug release; and an SIL – which tremendously accelerates drug release.^18^,^19^ We anticipate that this monomer will find diverse use in polymer therapeutics and broader drug delivery applications.

The activated monomer and HPMA were co-polymerized *via* the reversible addition–fragmentation chain transfer (RAFT) mechanism, Fig. 1C.^20^,^21^ We observed that conversions in these polymerizations were rather low, a likely explanation being a small release of 4-nitrophenol inhibiting the polymerization. Nevertheless, polymers were successfully obtained in a range of molar masses and content of the “activated” monomer. Conjugation of PANO to a polymer *via* a polymer-analogous reaction was first investigated *via* an *in situ* NMR monitoring which revealed a progressive conjugation reaction which was complete (with resolution of this technique) within 7 h (Fig. 2). This synthetic route afforded polymers with narrow dispersity (*Ð* < 1.3), molar mass from 6 to 13 kDa, and PANO content from 6 to 11 mol%.

**Fig. 2:**
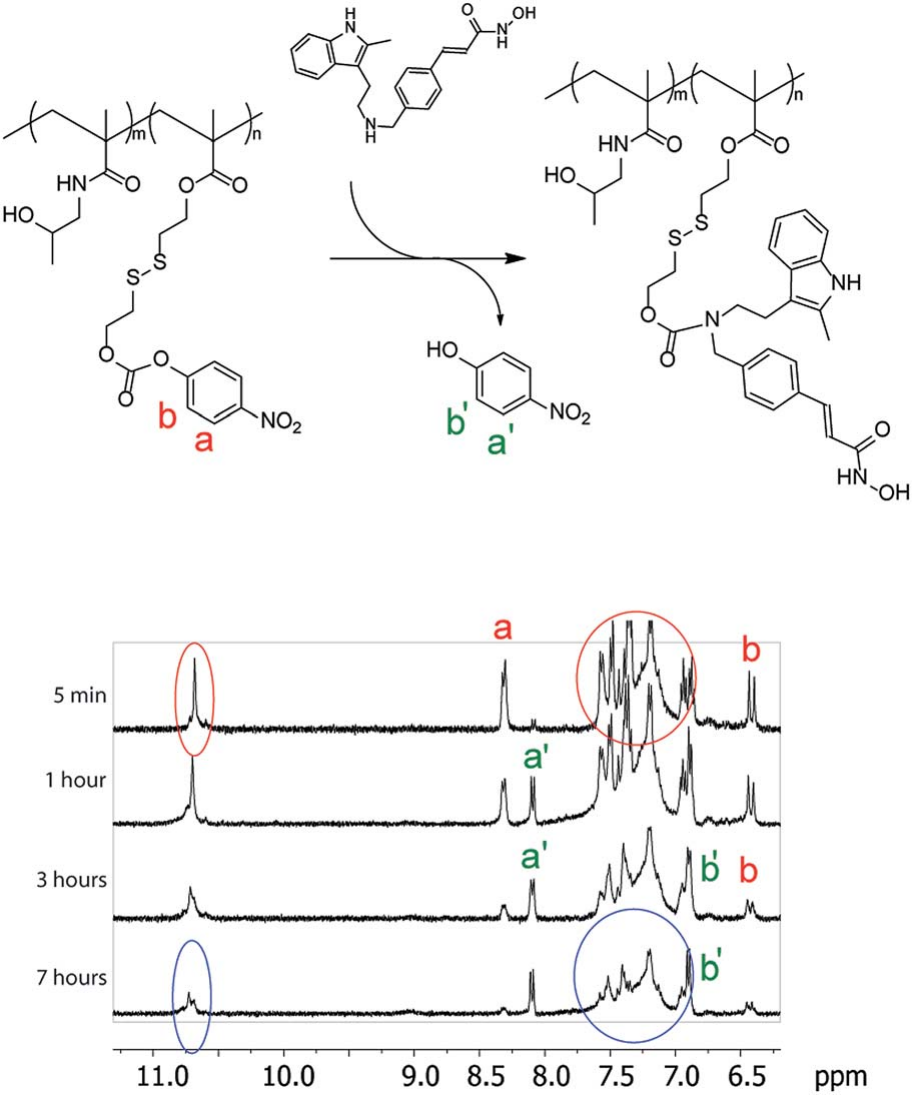
*In situ* ^1^H NMR monitoring of panobinostat conjugation to the polymers. A shift in proton signals (a to a′ and b to b′) illustrates the release of 4-nitrophenol, whereas broadening of peaks corresponding to panobinostat (6.9–7.7 ppm and 10.7 ppm) is a phenomenon characteristic of polymer conjugation.

Release of PANO from MP was studied using HPLC, Fig. 3. We have previously shown that the SIL linkage employed in this work remains stable at physiological conditions and undergoes a fast degradation upon a treatment with dithiothreitol or a natural intracellular thiol containing tripeptide, glutathione.^18^,^19^ In full agreement with this, PANO containing polymers showed only a negligible spontaneous drug release in physiological buffers but readily released PANO under reducing conditions (Fig. 3).

**Fig. 3:**
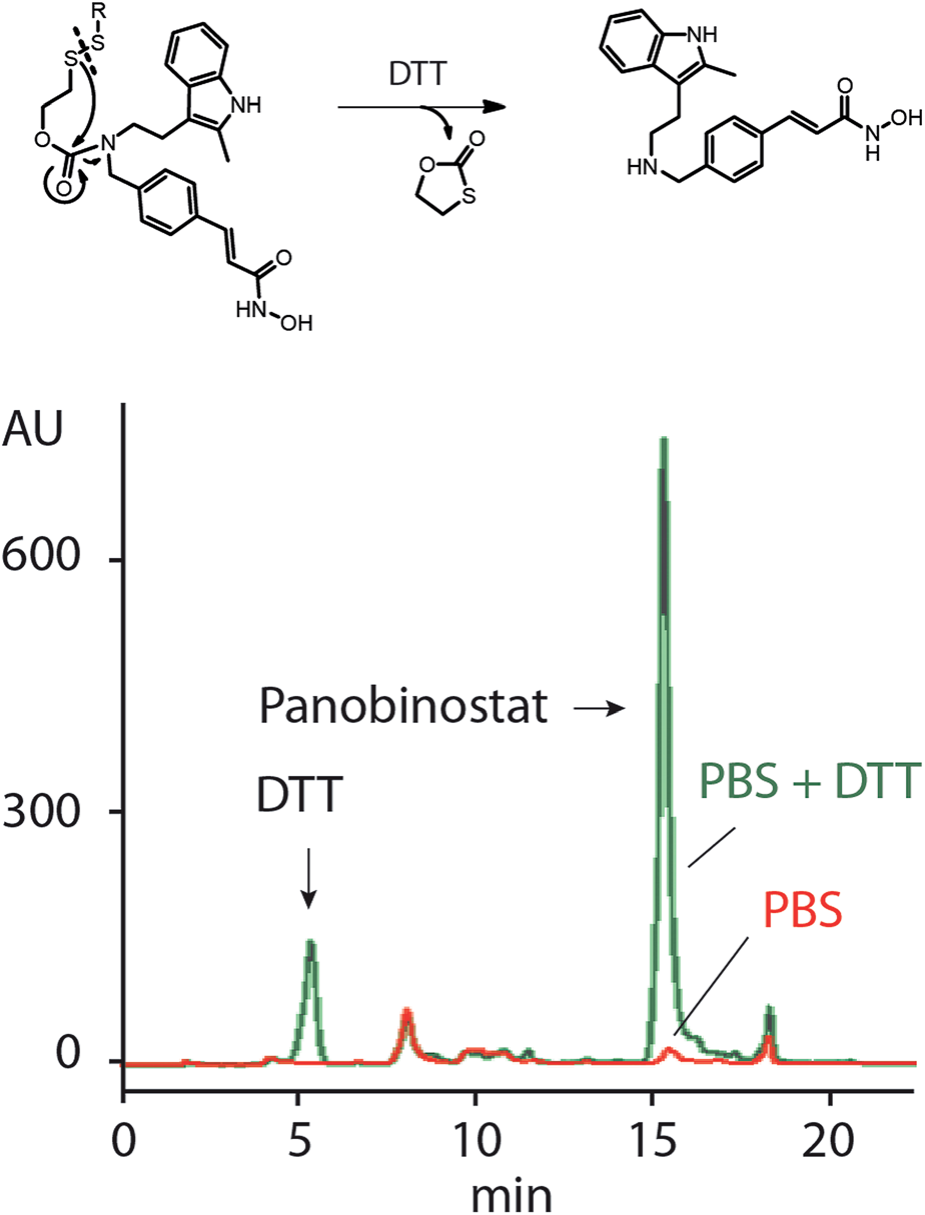
Schematic illustration of release of panobinostat from MP upon reduction with DTT (top) and HPLC data illustrating release of PANO from MP under reducing conditions (5 mM DTT). PBS stands for phosphate buffered saline (pH 7.4).

The utility of the MP containing PANO as LRA against HIV was first investigated in ACH2 and U1 cell models (T cell line and promonocyte cell line respectively) latently infected with HIV-1 by incubating the cells with the polymers at concentration 1 mg L^–1^. Pristine PHPMA revealed no activity as a latency reversing agent, Fig. 4. In contrast, MPs proved to be highly efficacious and for both cell lines, optimal compositions of MP matched the efficacy of 100 nM PANO (being therapeutically relevant concentration) at no expense to the cell toxicity of treatment. With regard to the optimal molar mass of the MP, no clear trend emerged from this dataset, most likely due to narrow range of studied molar mass. With regard to the MP drug loading, 11 kDa polymer sample with double the amount of conjugated drug was markedly more effective in activating the latent virus (see data for U1 cell line). EC_50_ value for MP (13 kDa, 6% drug loading) was established at 1 mg L^–1^ for both cell lines (for titration curves, see ESI Fig. SI5†). Expressed in mole equivalents of PANO, the polymers were ∼10-fold less potent than the pristine drug. This pharmacodynamic loss is not unexpected for polymer therapeutics^22^,^23^ and is due to *e.g.* decreased cell entry of MP compared to the free drug as well as incomplete release of the drug from the carrier, whereas the overall benefit of MP is typically due to optimization of pharmacokinetics of the drug on systemic and sub-cellular levels.^5^,^6^

**Fig. 4:**
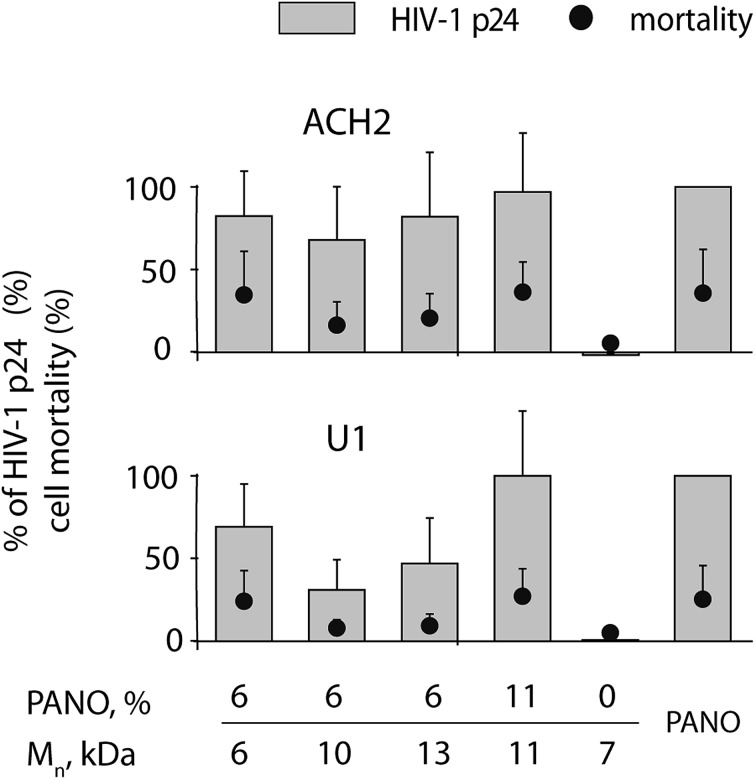
HIV-1 expression in U1 and ACH2 cells treated with panobinostat conjugated to PHPMA at 1 mg L^–1^ conc. for 48 h; pristine PANO at conc. 100 nM and pristine PHPMA at conc. 10 mg L^–1^ were used as controls. Data are present as mean of at least 3 independent experiments ±SD in % response compared to 100 nM pristine panobinostat.

Further characterization of the anti-latency treatment using MP was performed using a J-Lat full length clone 6.3 cell line which is a Jurkat derived cell line infected with a retroviral construct consisting of full length HIV-1 genome without *env* and *nef*, but containing eGFP (green fluorescent protein).^24^ eGFP is being co-expressed together with the HIV genome upon reactivation from latency. The green fluorescence of the cells can be quantitatively tracked with flow cytometry in parallel to the live/dead assay, Fig. 5. The two ways to activate the virus – using MP or pristine PANO – were near identical in terms of fraction of cells becoming positive for eGFP (in each data set – top left quadrangle) as well as the associated toxicity effect (bottom right quadrangle) – illustrating the success in the design of MP. Curiously, flow cytometry analysis revealed that toxicity of treatment with both MP and PANO agents was not due to activation of HIV and dead cells were devoid of the HIV-associated fluorescence. Upon a treatment with PANO or MP, a population of cells with activated HIV was not stained with a reagent for cell death, whereas the cells staining positive for cell death were devoid of activated HIV. This observation may present significant novelty for the rapidly developing field of HIV anti-latency treatment; we are now investigating this in detail.

**Fig. 5:**
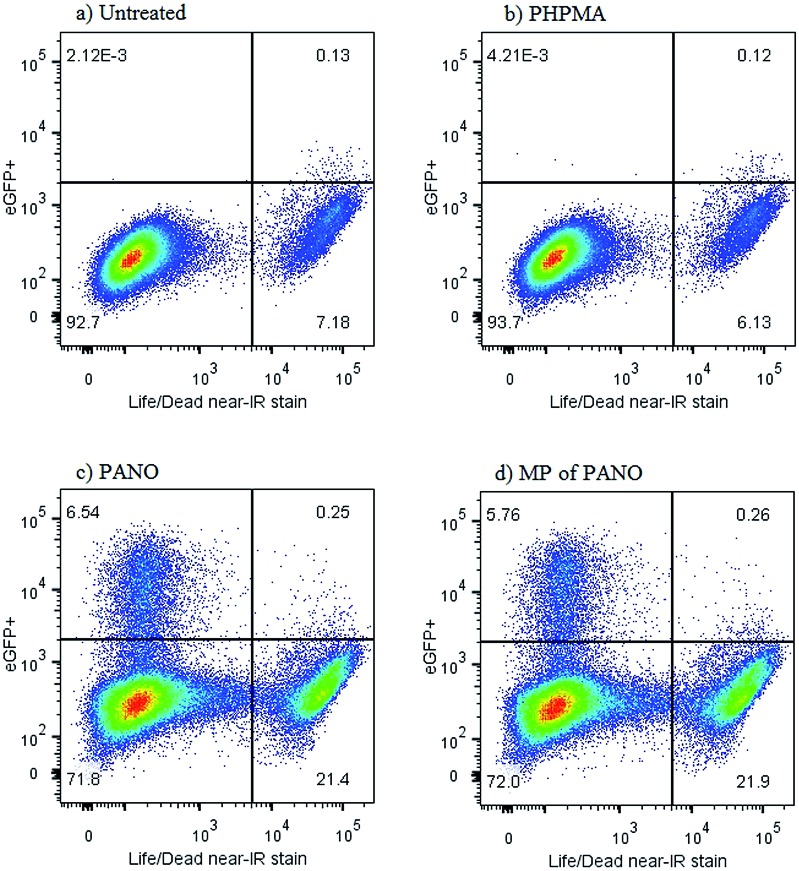
Viability and HIV expression in non-treated J-Lat cells (a) or J-Lat cells incubated with (b) pristine PHPMA polymer at conc. 10 mg L^–1^ (c) pristine panobinostat at conc. 50 nM, (d) PHPMA 6.4% PANO, conc. 10 mg L^–1^.

Earlier we have explored the broader impact of PANO on immunomodulation.^25^,^26^ We identified a small but significant increase in T cell activation by PANO, an effect that may be significant for the drugs' ability to turn on transcription of integrated proviruses due to a more favorable cellular milieu. Therefore, we sought to determine the activation of T cells by MP through quantifying the expression of the early T cell activation marker CD69. The activation of human CD4+ T cells by MP of PANO was comparable to or higher than activation by pristine PANO used at 50 nM concentration, Fig. 6. Importantly, no cell death above background was detected in any of the samples as determined by flow cytometry. The highest expression of CD69 was achieved with MPs of PANO was at 11.1 mg L^–1^ at which concentration activation of T cells was comparable or exceeding that achieved by the pristine drug. Pristine polymer PHPMA did not induce any activation of T cells clearly indicating that activity of MPs lies primarily in the release of drug itself.

**Fig. 6:**
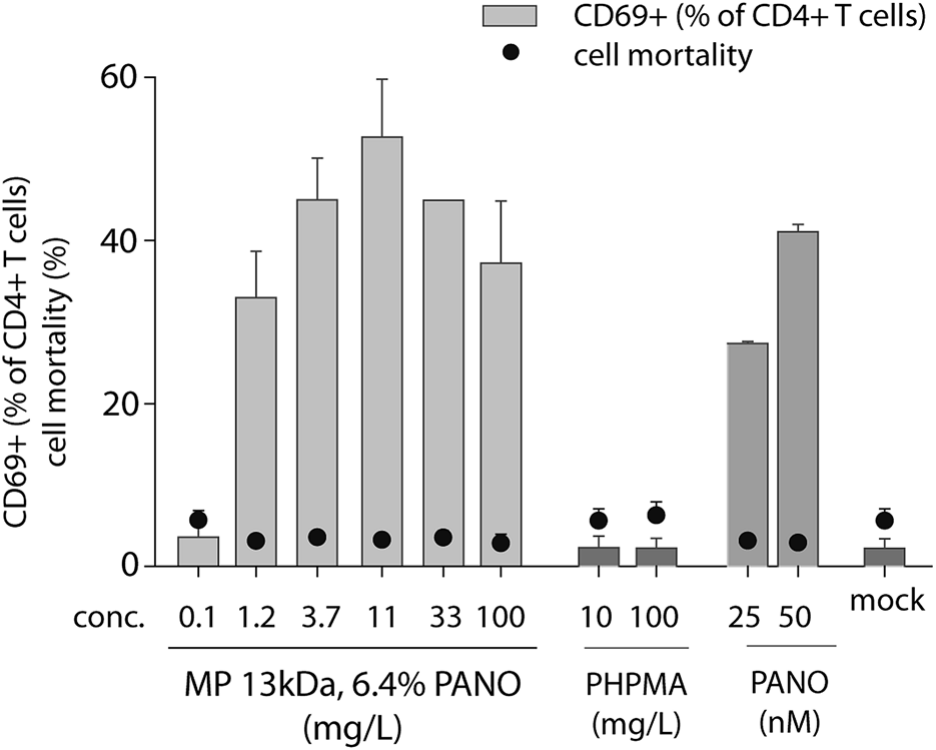
Expression of early marker of activation CD69 in CD4+ human T cells incubated for 18 h with serial dilutions of PHPMA 13 kDa, 6.4% PANO, pristine PANO at concentration 25 ad 50 nM and PHPMA at conc. 10 and 100 mg L^–1^. The results are an average of CD69+ expression in 2 donors.

Taken together, the results presented above establish a novel bioconjugation approach to create MP of PANO, an HDACi agent with virtually no arms for conjugation. Resulting polymers were equi-efficacious to the parent drug in terms of net virus activation and CD4+ T cell activation. We expect that these results would lead to the advent of MP and ADC for this and other HDACi with similar, highly challenging for conjugation structure.

## Materials and methods

### Chemicals

All chemicals were bought from Sigma-Aldrich and used without further purification, unless stated otherwise. Panobinostat was synthesized according to the Novartis patent,^27^ with the synthesis of 4-formyl cinnamic acid made as described in patent^28^ and 2-methyl tryptamine made as described in literature.^29^

### Synthesis of polymer prodrugs

The synthesis of the activated monomer, the subsequent copolymerization and the postfunctionalization is described in detail in the ESI.† In short, the activated monomer, HPMA, the initiator and the RAFT agent were dissolved in DMSO in an ampoule. The reaction mixture was degassed by four freeze–pump–thaw cycles or until gas no longer appeared during the thaw cycle. The reaction mixture was allowed to polymerize at 70 °C. The ampoules were opened and the polymer precipitated into diethyl ether, filtered and washed with an acetone–ether mixture of 1 : 1. Panobinostat was conjugated to the activated polymer by dissolving the polymer in DMSO and adding equimolar panobinostat with respect to the pendant groups. The conjugate was recovered by carefully extracting the DMSO with diethyl ether and triturating the polymer with a 1 : 1 acetone ether mixture.

### Polymer analysis

Size-exclusion chromatography (SEC) was performed using a system comprising a LC-20AD Shimadzu HPLC pump, a Shimadzu RID-10A refractive index detector and a DAWN HELEOS 8 light scattering detector along with a SPD-M20A PDA detector, equipped with an Mz-Gel SDplus Linear column, with 5 μm particles a length of 300 mm and an internal diameter of 8 mm from MZ-Analysentechnik, providing a molecular weight range of 1000–1 000 000. This column was used with DMF with 10 mM LiBr at 30 °C and a flow-rate of 1 mL min^–1^. The *dndc* of 0.1 used in the analysis was measured from an HPMA homopolymer, assuming full mass recovery. Nuclear magnetic resonance (NMR) spectra were obtained with a Varian Mercury 400 MHz NMR spectrometer.

### Cell culture

U1, ACH2, J-Lat Full Length Clone 6.3 cells (all obtained through the NIH AIDS Reagent Program) and human Peripheral Blood Mononuclear Cells (PBMCs) were maintained in complete RPMI media (RPMI1640 (Lonza, Basel, Switzerland) containing 2 mM l-glutamine, 50 U ml^–1^ penicillin, 50 μg ml^–1^ streptomycin and 10% heat-inactivated FCS (Invitrogen, Glostrup, Denmark)). Cells were grown on T75 bottles (Nunc, Roskilde, Denmark) at 37 °C with 5% CO_2_.

### Stimulation of latently infected cell lines

U1 cells and ACH2 were seeded at initial density of 2 × 10^5^/well in a 96-well format in 100 μL of complete RPMI media. Compounds were added at desired concentrations in 100 μL media. Identical dimethyl sulfoxide (DMSO) levels were used for untreated control. All stimulations were performed in triplicate. 42 h later supernatants were harvested and left for inactivation in 1% Empigen for 45 min. Levels of HIV-1 p24 gag were determined by ELISA. IC_50_ values were calculated based on titration curves using GraphPad Prism 6. Cell viability was measured by staining with Live/Dead near-IR (Invitrogen, Denmark) as follows: cells were harvested, washed with PBS, and stained with Live/Dead near-IR dye for 30 min on ice. Then cells were washed with PBS, fixed in 1% formaldehyde solution and analyzed by flow cytometry on BD FacsVerse. Data was analyzed using FlowJo Version 10.0.7.

J-Lat cells are Jurkat derived cells virally infected with retroviral construct: HIV-R7/E-/GFP; full length HIV-1 without *env* and *nef*, which means that GFP (green fluorescent protein) is expressed together with HIV genome upon reactivation from the state of latency. The green fluorescence of the cells can be tracked by flow cytometry. J-Lat cells were seeded at initial density of 1 × 10^6^ cells per well in a 24-well format in 500 μL of complete RPMI media. Compounds were added at desired concentrations in 500 μL media. Identical dimethyl sulfoxide (DMSO) levels were used for untreated control. 24 h later cells were harvested and stained with Life/Dead near-IR (Invitrogen, Denmark) and then fixed in 1% paraformaldehyde solution. Expression of GFP and cell viability were analyzed by flow cytometry on BD FACSVerse. Data was analyzed using FlowJo Version 10.0.7 (TreeStar, USA).

### Early activation of human CD4+ T cells

The blood used for PBMC isolation was obtained from healthy volunteer blood donors in Central Region Midt, Denmark covered by an ethical permission obtained from the Regional Research Ethics Committee. All participating blood donors signed an informed consent form prior to blood donation. Human PBMCs were isolated from two healthy donors using Ficoll gradient. Cells were seeded in a 48-well plate at density of 1 × 10^6^ cells per well. After 2 hours drugs diluted in complete RPMI media were added at indicated concentrations and incubated with cells in final volume of 500 μL of complete media for 18 hours. Subsequently cells were harvested and stained for flow cytometry. Every sample was divided into two tubes, from which one was stained with APC CD69 antibody, and the other one with isotype control APC IgG1, κ. Cells were washed in PBS and then stained with Live/Dead stain (Invitrogen, Denmark) for 30 min on ice. Cells were then washed two times in PBS. Surface blocking was performed by adding 5 μL of FcBlock (Sigma, Denmark) and incubating with cells for 10 min on ice. Subsequently surface staining was performed with 5 μL FITC CD3, 1 μL Pe-Cy7 CD4 (clone SK3), 1.25 μL APC CD69 (all obtained from BioLegend) for 30 min at room temperature. Finally cells were washed with FACS buffer and analyzed on BD FACSVerse. Data was analyzed using FlowJo Version 10.0.7. Gating strategy was performed as described previously.^3^

## Supplementary Material

Supplementary information
