# Supplementary material for: HIV anti-latency treatment mediated by macromolecular prodrugs of histone deacetylase inhibitor, panobinostat
Source: Chem Sci. 2016 Jan 5;7(3):2353–8. doi: 10.1039/c5sc03257a (PMC6003675; doi:10.1039/c5sc03257a)
Supplement: Supplementary file 1 [file SC-007-C5SC03257A-s001.pdf]

Electronic Supplementary Material (ESI) for Chemical Science.
This journal is © The Royal Society of Chemistry 2016

Supporting information

HIV anti-latency treatment mediated by macromolecular prodrugs of histone deacetylase inhibitor, panobinostat

Kaja Zuwala,1,2 ^ Anton A.A. Smith,1^ Martin Tolstrup,2* Alexander N. Zelikin1,3*

Synthesis of activated SIL monomer

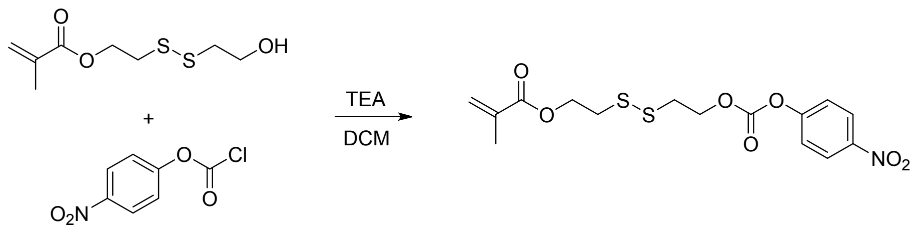

2-((2-hydroxyethyl)disulfanyl)ethyl methacrylate was made as described previously. [1]

Experimentals: p-nitrophenol chloroformate (398 mg, 1.98 mmol) in dichloromethane (DCM) (2.5 mL) was added to a solution of 2-((2-hydroxyethyl)disulfanyl)ethyl methacrylate (400 mg, 1.80 mmol) and triethylamine (TEA) in DCM (2.5 mL) over an ice bath. After addition the ice bath was removed and the reaction turned yellow and cloudy. After 50 minutes, thin layer chromatography (TLC) (DCM) showed depletion of methacrylate disulphide. The reaction was quenched with NH_4Cl sat (30 mL), washed with brine (40 mL), and the aqueous phases extracted with DCM (10 mL). The organic phases collected, dried over Na_2SO_4 and concentrated *in vacuo* to yield a yellow oil. The crude product was purified on silica, eluting from 3:7 to 0:1 heptane:DCM as a yellow oil (499 mg, 1.29 mmol, 72% yield).

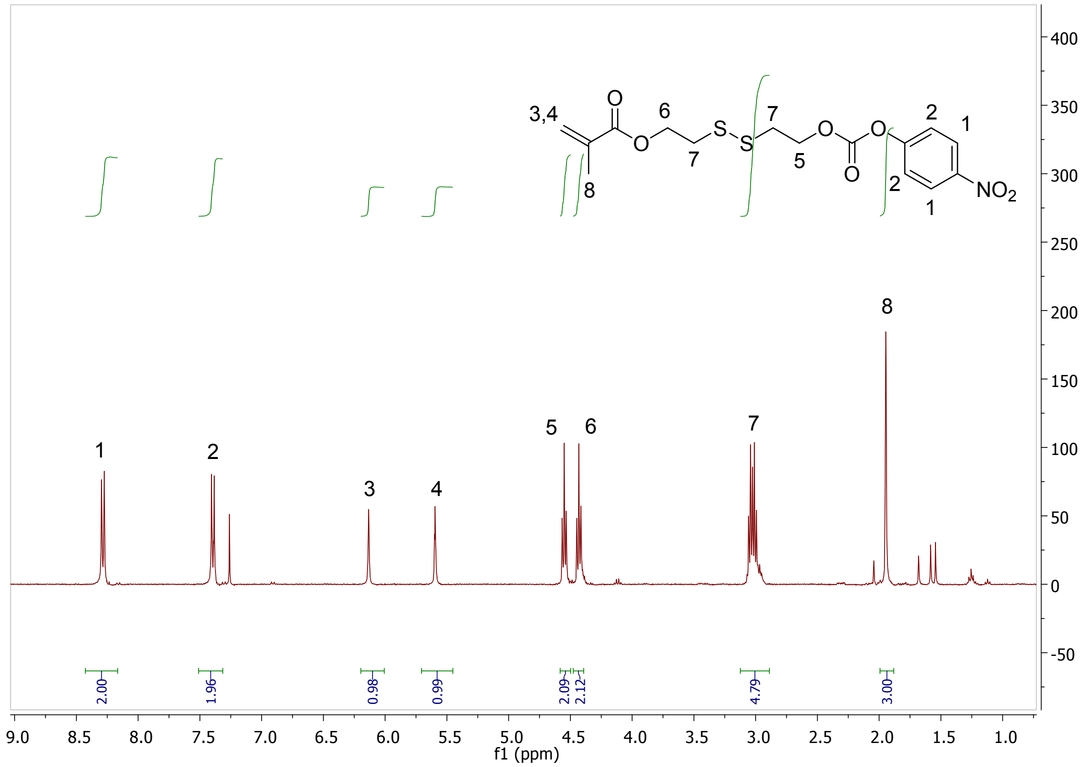

H [1]NMR (400 MHz, $CDCl_3$) δ 8.29 (d, *J* = 9.2 Hz, 2H, H1), 7.40 (d, *J* = 9.2 Hz, 2H, H2), 6.13 (s, 1H, H3), 5.60 (s, 1H, H4), 4.55 (t, *J* = 6.5 Hz, 2H, H5), 4.43 (t, *J* = 6.6 Hz, 2H, H6), 3.08 – 2.95 (m, 4H, H7), 1.95 (s, 3H, H8).

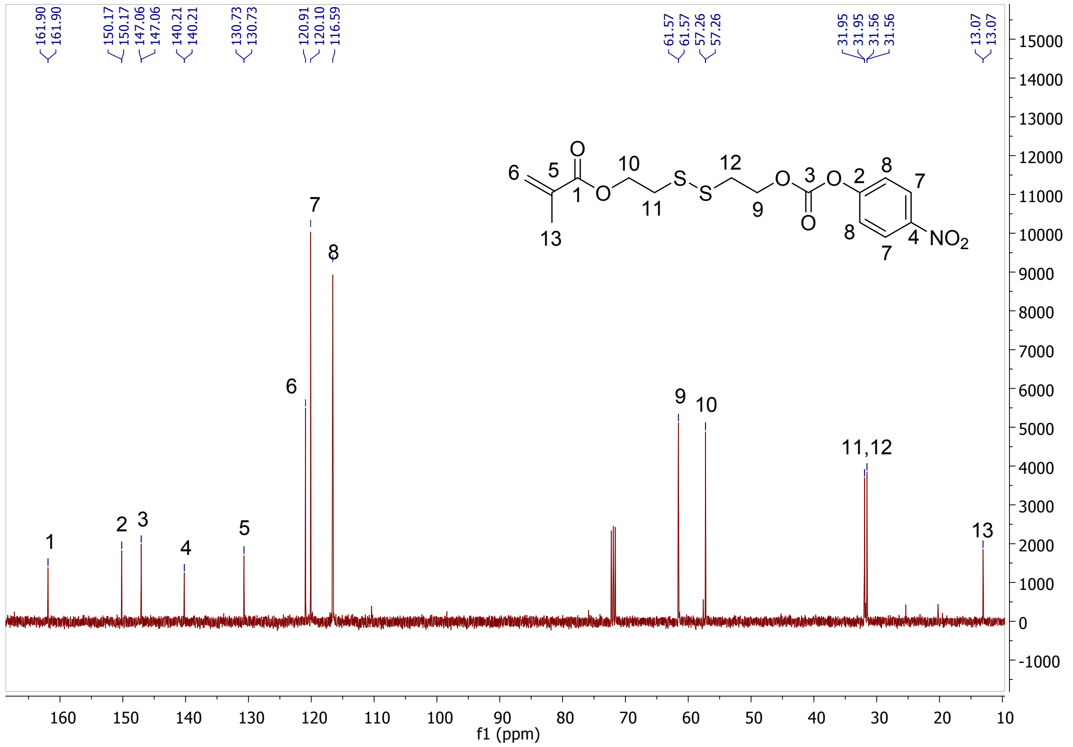

^{13}C NMR (100 MHz, $CDCl_3$) δ 161.90 (C1), 150.17 (C2), 147.06 (C3), 140.21 (C4), 130.73 (C5), 120.91 (C6), 120.10 (C7), 116.59 (C8), 61.57 (C9), 57.26 (C10), 31.95 (C11), 31.56 (C12), 13.07 (C13).

HRMS (ESI+) *m/z* calcd for $C_{15}H_{18}NO_7S_2^+$ [M+H$^+$]= 388.05192 found 388.0522 $C_{15}H_{17}NO_7S_2Na^+$ [M+Na$^+$]= 410.03386 found 410.0339.

Polymerizations

Polymer D

An ampoule was charged with *N*-(2-hydroxypropyl)methacrylamide HPMA (286 mg, 2.00 mmol), MM (41 mg, 1.1 mmol), 4,4'-azobis(4-cyanovaleric acid) (ACVA) (0.89 mg, 3.2 µmol) and 4-Cyano-4-[(dodecylsulfanylthiocarbonyl)sulfanyl]pentanoic acid (5.1 mg, 13 µmol) to a [HPMA+MM]/[RAFT] of 167 and a [RAFT]/[initiator] of 4. 0.29 mL dimethylsulphoxide (DMSO) was added and the ampoule was degassed with a minimum of four freeze-pump-thaw cycles on a vacuum line. The ampoule was subsequently flame sealed and left to react at 70° C for 16 hours.

The ampoules were opened and the polymer precipitated into diethyl ether, filtered and washed with an acetone-ether mixture of 1:1.

The polymer had a conversion of 18% corresponding to a calculated M_n of 5.0 kDa. The mass was measured to 10.8 kDa by Multi Angle Light Scattering Gel Permeation Chromatography (MALS-GPC) with a Đ of 1.178. The content of MM was measured by ^{1}H-NMR to be 11% of the total degree of polymerization (DP) by the intensity of the 4-nitrophenol at 8.3 ppm in comparison to the HPMA signal at 3.7 ppm

Polymer A, B and C

Polymer A, B and C were made with the following changes to the protocol for A

HPMA (143 mg, 1.00 mmol), MM (9.9 mg, 26 µmol), RAFT agent (A: 6.2 mg,15 µmol B: 3.1 mg, 7.7 µmol C: 1.4 mg, 3.4 µmol) ACVA (A: 1.1 mg,3.8 µmol B: 0.54 mg, 1.9 µmol C: 0.2 mg, 0.8 µmol) in 0.14 mL of DMSO each.

B: 5.8 kDa GPC, 4.6 kDa calc (46% conversion). Dispersity of 1.032, elution tailing into the solvent peak. This obstructs the analysis and causes the dispersity to be falsely narrow. MM content was 5%
C: 10.1 kDa GPC, 7.0 kDa calc (35% conversion). Dispersity of 1.073, MM content was 5%
D: 13.0 kDa GPC, 11.0 kDa calc (24% conversion) Dispersity of 1.104, MM content was 6%

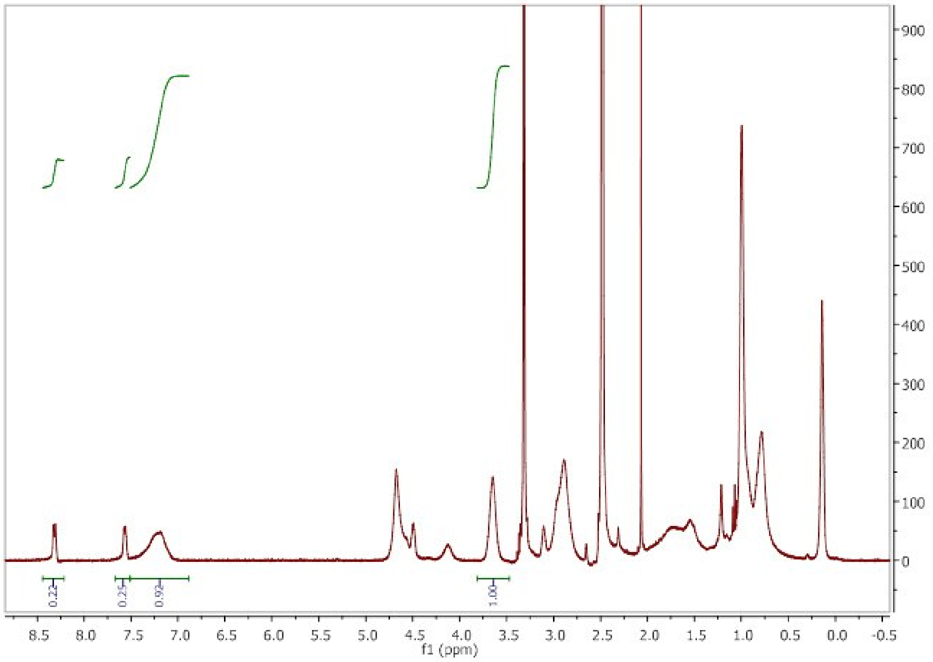

Figure SI1 : Proton NMR of MM and HPMA copolymer

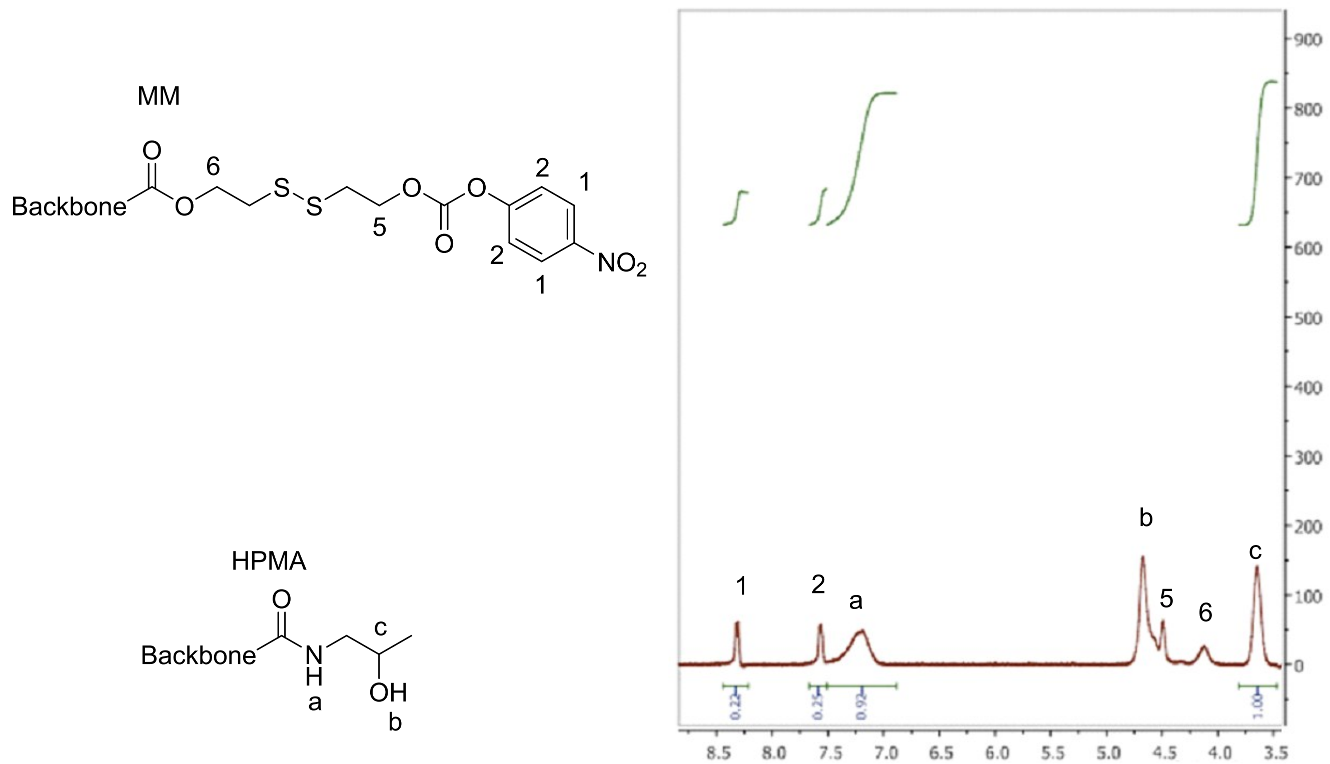

Figure SI2 : Proton NMR of MM and HPMA copolymer. MM content was calculated by comparing integrals of the HPMA signal at 3.7 ppm (c) and the signals from signals at 8.3 from MM (1).

Conjugations with panobinostat to polymers A, B and C

40 mg was weighed out of the polymers A,B and C, equivalent to 12.1 umol activated pendant groups. Each were dissolved in 0.4 mL deuturated DMSO. Panobinostat (4.6 mg 13.2 umol M=349.4 g/mol) was dissolved in 0.1 mL DMSO-d6 and added to each of the solutions and was allowed to react for 7 hours at rt and then left at 5° C overnight. The polymer conjugates were purified by phase extraction of the DMSO into ether, followed by trituration of the polymer in ether and acetone.

The panobinostat conjugation to D was performed in a similar manner, but with 10 mg panobinostat added instead to accommodate for the higher MM content of D.

These reactions yielded the polymers used in the *in vitro* assay in figure 4, with the panobinostat conjugates of A, B, C and D appearing from left to right.

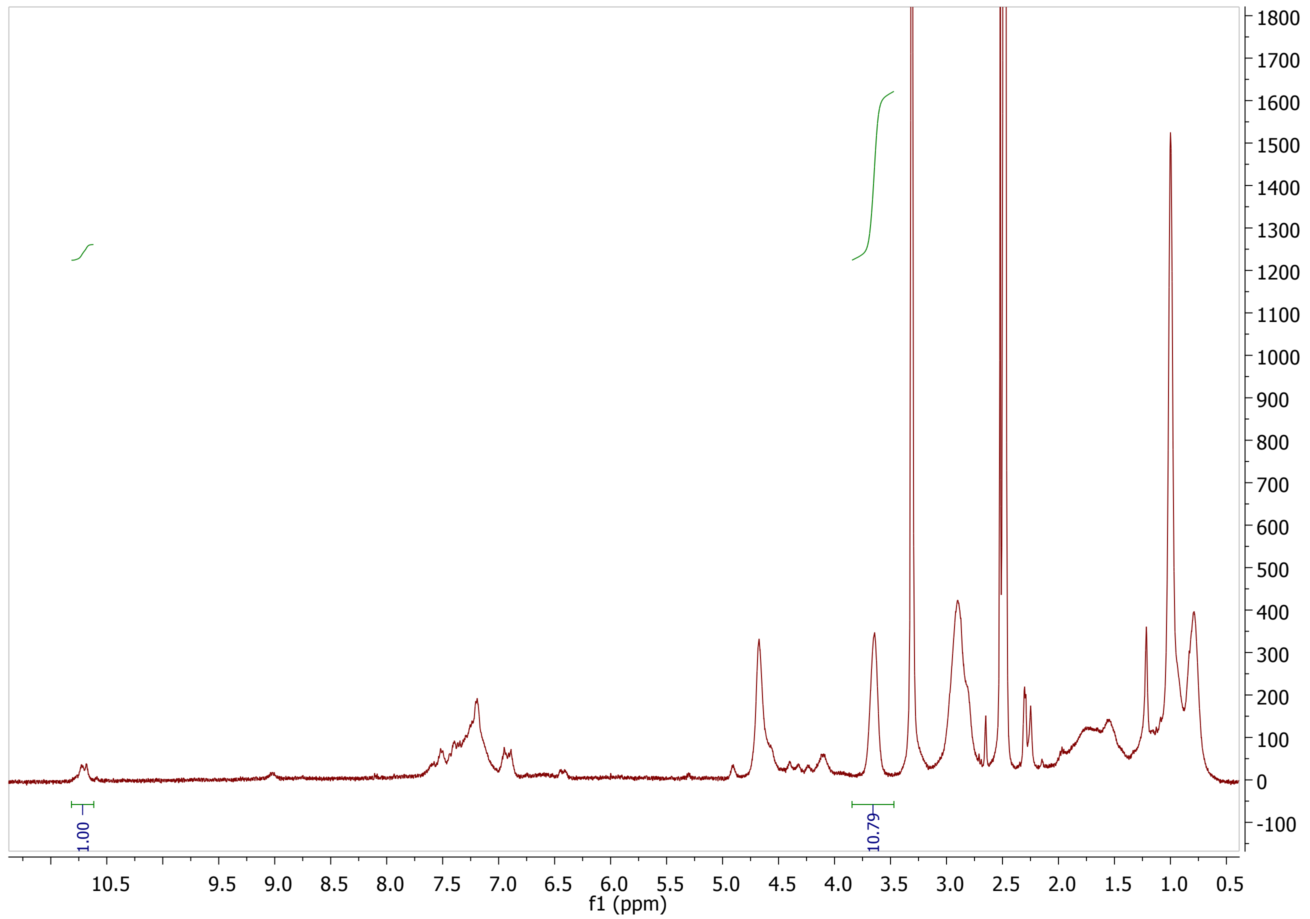

Figure SI3 : NMR analysis of copolymer D after panobinostat attachment.

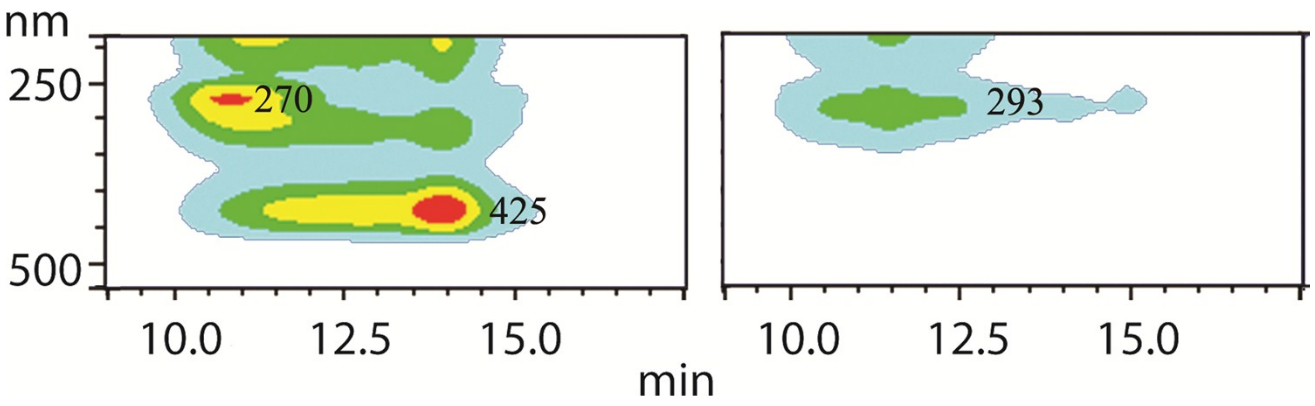

Figure SI4 : PDA elugram of Polymer D before (left) and after (right) panobinostat attachment. Before the conjugation there is a pronounced absorption of 4-nitrophenol both attached (270 nm) and released (425 nm)

as the unstable pendent group tails off the polymer peak from about 10 min. This non-specific release of 4-nitrophenol was only observed in the GPC and is contributed to the residual dimethylamine in the eluent DMF displacing 4-nitrophenol. No release of 4-nitrophenol was observed in NMR samples in the absence of a nucleophile.
After attachment the absence of 4-nitrophenol is noted, and a new max absorption is noted at 292 nm, corresponding to an absorption of panobinostat in DMF.

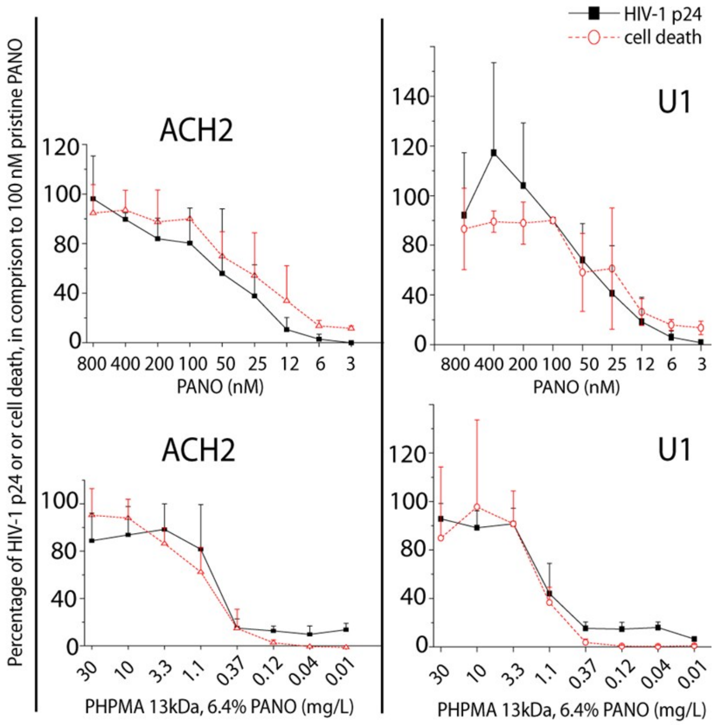

Figure SI5 :Dose response curves presenting level of expressed HIV-1 p24 protein in latently infected ACH2 and U1 cell line. Cells were preincubated with indicated concentrations of the pristine PANO or PHPMA 13 kDa, 6.4% PANO for 42 hrs. Then supernatants were harvested and the level of HIV-1 p24 protein was determined by ELISA p24. Cell viability was determined by staining with Life/Dead Near-IR stain (Invitrogen, Denmark) and analyzed by flow cytometry. All results were compared to the sample treated with 100 nM PANO. 100 nM concentration was chosen as the lowest one, which induces maximum expression of latent HIV in ACH2 and U1 cell line

1 Kock, A. *et al.* Disulfide reshuffling triggers the release of a thiol-free anti-HIV agent to make up fast-acting, potent macromolecular prodrugs. *Chem Commun* **50**, 14498-14500, doi:10.1039/c4cc04280h (2014).
